# Characterization of Phenotypes and Functional Activities of Leukocytes From Rheumatoid Arthritis Patients by Mass Cytometry

**DOI:** 10.3389/fimmu.2019.02384

**Published:** 2019-10-18

**Authors:** Adrien Leite Pereira, Samuel Bitoun, Audrey Paoletti, Gaetane Nocturne, Ernesto Marcos Lopez, Antonio Cosma, Roger Le Grand, Xavier Mariette, Nicolas Tchitchek

**Affiliations:** ^1^CEA – Université Paris Sud 11 – INSERM U1184, Immunology of viral infections and autoimmune diseases, IDMIT Infrastructure, Fontenay-aux-Roses, France; ^2^Department of Rheumatology, Hôpital Bicetre, Le Kremlin-Bicêtre, France; ^3^Center for Immunology of Viral Infections and Autoimmune Diseases, INSERM U1184, Paris-Sud University, Le Kremlin Bicêtre, France

**Keywords:** rheumatoid arthritis, mass cytometry, TLR stimulation, neutrophils, T-cells

## Abstract

**Background:** Rheumatoid arthritis (RA) is the most common autoimmune rheumatic disease and leads to persistent chronic inflammation. The pathophysiology of the disease is complex, involving both adaptive and innate immunity. Among innate immune cells, neutrophils have been rarely studied due to their sensitivity to freezing and they are not being collected after Ficoll purification.

**Methods:** We used mass cytometry to perform a multidimensional phenotypic characterization of immune cells from RA-treated patients, which included the simultaneous study of 33 intra- or extra-cellular markers expressed by leukocytes. We were able to focus our study on innate immune cells, especially neutrophils, due to a specific fixation method before freezing. In addition, blood samples were stimulated or not with various TLR agonists to evaluate whether RA-dependent chronic inflammation can lead to immune-cell exhaustion.

**Results:** We show that RA induces the presence of CD11b^low^ neutrophils (33.7 and 9.2% of neutrophils in RA and controls, respectively) associated with the duration of disease. This subpopulation additionally exhibited heterogeneous expression of CD16. We also characterized a CD11a^high^ Granzyme B^high^ T-cell subpopulation possibly associated with disease activity. There was no difference in cytokine expression after the stimulation of immune cells by TLR agonists between RA and controls.

**Conclusion:** Mass cytometry and our fixation method allowed us to identify two potential new blood subpopulations of neutrophils and T-cells, which could be involved in RA pathology. The phenotypes of these two potential new subpopulations need to be confirmed using other experimental approaches, and the exact role of these subpopulations is yet to be studied.

## Introduction

Rheumatoid arthritis (RA) is the most common inflammatory autoimmune rheumatic disease, with a prevalence of 0.5–1% in the adult population (1). RA is characterized by symmetric polyarticular inflammation of the joints, potentially leading to pain, deformity, and disability, resulting in major personal and societal costs (2, 3).

As RA is an autoimmune disease, most pathophysiology studies have focused on T and B-cells. However, it is also clear that innate immunity plays a role in shaping adaptive immunity, and much attention has been recently given to the study of monocytes and macrophages in RA (4). Neutrophils are numerically the most predominant innate immune cells both in blood and synovium. They have been studied *in vitro* for their capacity to form extracellular trap formation (NETosis) (5), playing a central role in the exposition of citrullinated auto-antigens (6). Indeed, the only new RA therapy currently in phase 3 trial is based on an anti-GM-CSF antibody, which targets both monocytes/macrophages and neutrophils (7). However, even though neutrophils play a very important role in RA, they have been poorly studied due to technical difficulties. Indeed, neutrophils are eliminated by Ficoll separation and are sensitive to freezing.

Mass cytometry is a single-cell technology that allows the simultaneous characterization of multiple leukocyte populations. Due to a method that we have developed to conserve cells before freezing, this technology also allows the study of all innate and adaptive immune cells, including granulocytes (8).

Here, our objective was to better characterize leukocytes in RA and discover new cell subsets specific to this disease through an unbiased approach. We compared cell populations from healthy donors and RA patients using a mass cytometry panel of 33 cell markers. We identified a potential specific CD11b^low^ CD16^high^ neutrophil subpopulation in RA patients, as well as a potential specific CD11a^high^ Granzyme B^high^ T-cell subpopulation. We also measured the response of leukocyte populations after stimulation with various TLR agonists to determine whether RA can lead to immune-cell exhaustion and render patients more susceptible to infectious diseases. Moreover, we demonstrated that RA does not affect TLR-dependent immune responses.

## Results

### Identification of Cell Populations by Mass Cytometry Profiling

Whole blood from nine RA patients (Table 1) and five healthy individuals were collected. Data concerning the age, previous and current treatment, as well as the presence of anti-cyclic citrullinated peptide antibodies (anti-CCP), rheumatoid factor (RF), and/or joint erosion were collected for each RA-treated patient. The group of nine RA patients was composed of two males and seven females. The age ranged from 36 to 85 years. The prescribed treatment regimens are shown in Table 1. Rituximab was prescribed for two RA patients and B-cell depletion was confirmed for both. The healthy-donor group was composed of three males and two females and the age ranged from 26 to 60 years. Characteristics of patients presented in Table 1 correspond to information collected at the time of blood sampling. Thus, only 3 RA patients were in active disease state (with a DAS28 score >3.2).

**Table 1:** Characteristics of rheumatoid arthritis patients.

**Patients**	**Age range (years)**	**Current treatments**	**Disease duration (months)**	**DAS28**	**Anti-CCP**	**Erosion**	**RF**
PAT-1	36–40	Prednisone	28	2.70	Yes	Yes	Yes
PAT-2	80–85	Tocilizumab	172	6.85	Yes	Yes	Yes
PAT-3	40–45	Nonsteroidal anti-inflammatory drugs	124	5.49	Yes	Yes	Yes
PAT-4	71–75	Tocilizumab	76	3.49	Yes	Yes	No
PAT-5	81–85	Etanercept, Methotrexate	136	2.68	Yes	Yes	NA
PAT-6	56–60	Methotrexate	76	1.89	NA	NA	NA
PAT-7	56–60	Methotrexate Hydroxychloroquine, Salazopyrine, Prednisone	148	2.58	Yes	No	Yes
PAT-8	50–55	Rituximab, Methotrexate	244	2.97	Yes	Yes	Yes
PAT-9	56–70	Rituximab, Methotrexate	304	2.56	Yes	Yes	Yes

*The age range, and previous and current treatments, as well as the presence of anti-cyclic citrullinated peptide antibodies (anti-CCP), rheumatoid factor (RF), and/or joints erosion indicated for each RA-treated patient. Characteristics of patients correspond to information collected at the time of blood sampling*.

Blood samples were labeled with a mass cytometry panel of 33 cell markers (Supplementary Table 1). This panel was designed to identify both innate and adaptive cell populations. To have a deep characterization of innate immunity processes, the antibody panel included phenotype, sensing, activation, costimulation, homing, adhesion markers, but also Fc receptors and inflammatory mediators.

**Figure 1:**
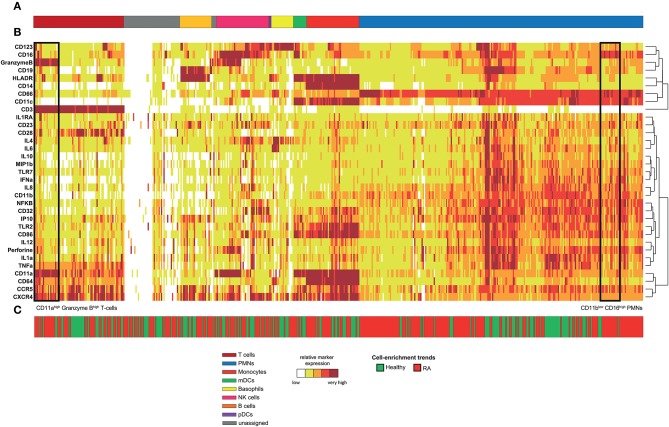
Phenotypic diversity of leukocytes in RA disease. Whole blood was collected from nine RA-treated patients and five healthy donors. Following red blood cell lysis, leukocytes of each sample were stained using a mass cytometry panel consisting of 33 markers. A SPADE analysis was performed to identify 500 cell clusters. A categorical heatmap, representing the relative marker expression for each cluster, was generated. Marker expression ranges were calculated based on the 5th and 95th percentile of the expression of each marker. Then, ranges were divided into five uniform categories. The categorization of marker expression was computed based on the means of the cell cluster expression medians of each marker. These categories represent negative, low, medium, high, and bright relative marker expression using a color scale ranging from white to dark red. Hierarchical clustering was performed to gather clusters with similar phenotypes. Two additional hierarchical clusterings, one for clustering markers and the other for non-clustering markers, were performed to visualize markers with similar co-expression patterns. **(A)** Cell clusters were manually annotated based on the expression of specific cell markers and are represented by different colors. **(B)** Representation of the heatmap. **(C)** Chart showing the cell-enrichment trend toward an RA or healthy profile for each cluster.

Following data acquisition, spanning-tree progression analysis of density-normalized events (SPADE) was performed (9). This analysis was parameterized to generate a SPADE tree composed of 500 cell clusters. The SPADE clustering was based on the expression levels of the 33 markers of innate and adaptive cell populations to capture maximal cell diversity. We interpreted the cell cluster phenotypes by generating a categorical heatmap representing the relative marker expression for each cluster (Figure 1). T-cell, B-cell, neutrophil, NK cell, monocyte, and dendritic-cell clusters were directly annotated according to their levels of CD3, CD11c, CD14, CD16, CD19, CD66, CD123, Granzyme B, and HLADR (Figures 1A,B).

### Rheumatoid Arthritis Induces High Neutrophil Diversity

The 500 identified clusters were classified into two categories according to their trends of cell cluster abundances (Figure 1C) between RA patients and healthy donors.

The first category (in red) corresponds to clusters for which the cell abundance was enriched in RA patients, whereas the second category (in green) corresponds to clusters for which the cell abundance was enriched in healthy donors. Strikingly, neutrophils represented 46.6% of the cell clusters, reflecting the high diversity of this population (Figure 1A, in blue). In comparison, the pDC population represented only 0.4% of the cell clusters. In addition, most of the cell clusters for each cell population were enriched (but not necessarily significantly) in the RA patients (63.2% of all the cell clusters), especially in neutrophils (68.7% of neutrophil clusters). Therefore, RA has a strong influence on the neutrophil compartment.

### Rheumatoid Arthritis Induces the Generation of a CD11b^low^ CD16^high^ Neutrophil Subpopulation Associated With the Duration of Disease

**Figure 2:**
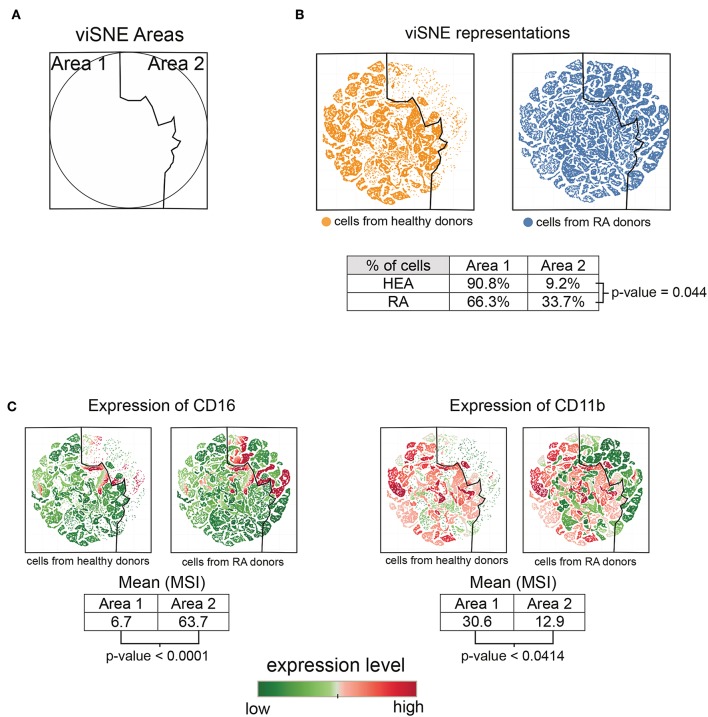
Characterization of the CD11b^low^ CD16^high^ neutrophil subpopulation. All cells contained in neutrophil clusters from the SPADE analysis were computationally isolated and represented using viSNE, based on the expression of CD11b and CD16. **(A)** viSNE representation showing two phenotypic areas delineated according to the localization of cells from healthy donors (Area 1) and RA patients (Area 2). **(B)** viSNE maps representing the distribution of neutrophils according to their expression of CD11b and CD16. **(C)** viSNE maps representing the levels of CD11b and CD16 expressed by neutrophils.

Based on the heatmap and T-cell cluster abundances (Figure 1), we identified a potentially new subpopulation of neutrophils expressing a low level of CD11b and high level of CD16 specifically enriched in RA patients (indicated at the bottom of the heatmap). We computationally isolated all neutrophils from the RA and healthy donors based on the SPADE tree to better characterize and represent this subpopulation. Then, we generated a viSNE representation to visualize the distribution of neutrophils according to their expression of CD11b and CD16 (Figure 2).

The phenotypic landscape of the neutrophils was divided into two areas, called viSNE Area 1 and viSNE Area 2 (Figure 2A). These two areas were delineated according to the localization of cells from healthy individuals and RA patients (Figure 2B). In total, 90.8% of cells from the healthy donors and 66.3% of cells from the RA patients were located in viSNE Area 1. Inversely, 9.2% of cells from the healthy donors and 33.7% of cells from the RA patients were located in viSNE Area 2. In addition, the cells from the RA patients were statistically more numerous in Area 2 than those from healthy donors (*p* = 0.044).

We next compared the means of signal intensities (MSI) of CD16 and CD11b between viSNE Area 1 and Area 2, which were enriched in healthy donors and RA patients, respectively. Cells from Area 2 showed lower levels of CD11b than those from Area 1 (*p* = 0.0414) (Figure 2C). In contrast, cells from Area 2 showed higher levels of CD16 than those of Area 1 (*p* ≤ 0.0001). This potentially new subpopulation of neutrophils with a CD11b^low^ CD16^high^ phenotype was not associated with the Disease Activity Score 28 (DAS 28) but significantly correlated (Spearman correlation coefficient = 0.7447; *p* = 0.0213) with the duration of disease (Supplementary Figure 1A).

Overall, these CD11b^low^ CD16^high^ neutrophils appear to be significantly enriched in RA patients relative to healthy donors and associated with the duration of disease. No significant correlations were identified with other clinical data.

### Rheumatoid Arthritis Induces the Generation of a CD11a^high^ Granzyme B^high^ T-Cells Subpopulation

**Figure 3:**
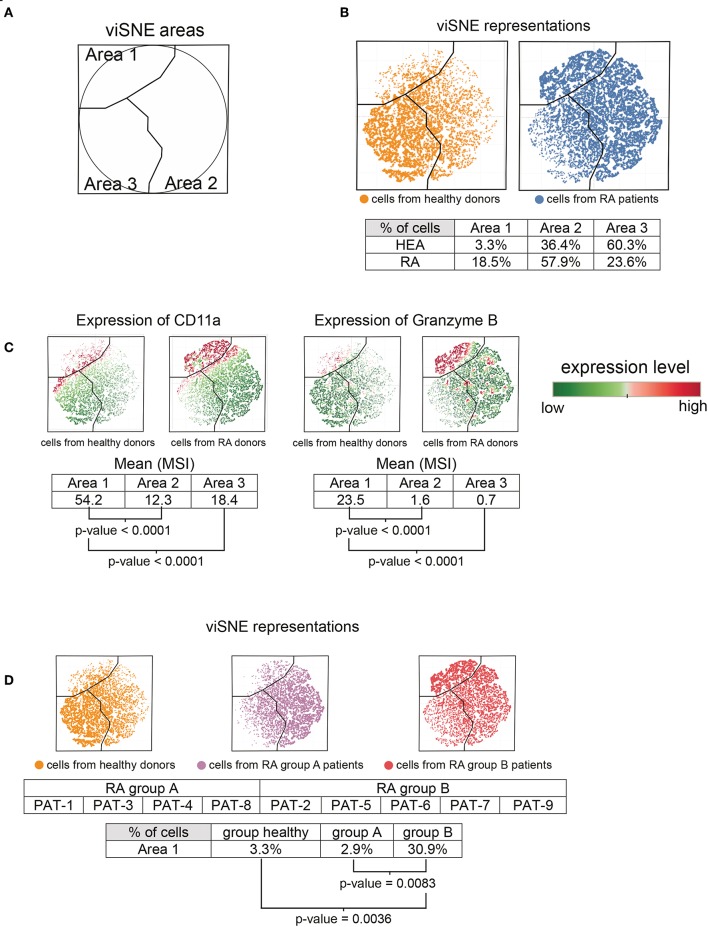
Characterization of CD11a^high^ Granzyme B^high^ T-cell subpopulation. All cells contained in T-cell clusters from SPADE analysis were computationally isolated and represented using viSNE, based on the expression of CD11a, CCR5, and Granzyme B. **(A)** viSNE representation showing three phenotypic areas delineated according to the localization of cells from RA patients (Areas 1 and 2) and healthy donors (Area 3). **(B)** viSNE maps representing the distribution of T-cells according to their expression of CD11a, CCR5, and Granzyme B. **(C)** viSNE maps representing the levels of CD11a and Granzyme B expressed by T-cells. **(D)** RA-patients were split into two groups, called group A and group B. viSNE maps representing the distribution of T-cells, according to their expression of CD11a, CCR5, and Granzyme B, for healthy donors and each group of RA patients.

Based on the heatmap and cell cluster abundances (Figure 1), we also identified a potentially new CD11a^high^ Granzyme B^high^ T-cell subpopulation (indicated at the bottom of the heatmap) that was specifically enriched in RA patients. We computationally isolated all T-cells from the RA patients and healthy donors from the SPADE tree to better characterize this subpopulation. Then, we generated a viSNE representation to visualize the distribution of T-cells according to their expression of CD11a, Granzyme B, and CCR5 (Figure 3).

The viSNE map was split into three areas, called viSNE Areas 1, 2, and 3 (Figure 3A). These three areas were delineated according to the localization of cells from healthy individuals and RA patients (Figure 3B). In total, 60.3% of cells from healthy donors and 23.6% of cells from RA patients were present in Area 3. However, the cell abundances in Area 3 between RA patients and healthy donors were not statistically different. Similarly, although Areas 1 and 2 were enriched in the RA patients (Area 1: 18.5%, Area 2: 57.9%) relative to the healthy donors (Area 1: 3.3%, Area 2: 36.4%), we observed no significant differences.

We next focused our analysis on cells from Area 1, in which the subpopulation of CD11a^high^ Granzyme B^high^ T-cells was enriched in RA patients (Figure 3C). Indeed, the levels (MSI) of CD11a and Granzyme B were significantly higher in Area 1 compared to Areas 2 and 3 (*p* < 0.0001 for each marker and each comparison). This specific subpopulation was absent from four RA patients (called group A patients, comprising patients 1, 3, 4, and 8), whereas it was present in five (called group B patients, comprising patients 2, 5, 6, 7, and 9) (Figure 3D). The percentage of CD11a^high^ Granzyme B^high^ T-cells in Area 1 was significantly higher in group B patients than that of healthy donors (*p* = 0.0036) or group A patients (*p* = 0.0083). The apparition of this specific subpopulation tended to negatively correlate (Spearman correlation coefficient = −0.6667, *p* = 0.0588) with the DAS 28 (Supplementary Figure 1B). Thus, this subpopulation seems to be more abundant in patients with a low-remission disease activity.

Moreover, the CD11a^high^ Granzyme B^high^ T-cells was significantly (*p* = 0.0440) higher in patients treated with methotrexate than those who received other treatments (Table 1). Indeed, three of five patients from group B were treated with methotrexate, vs. only one of four patients from group A.

### Monocytes and Dendritic Cells From Rheumatoid Arthritis Patients Respond Normally to TLR Stimulation

We evaluated whether RA-dependent chronic inflammation can lead to immune-cell exhaustion by stimulating leukocytes from RA patients and healthy donors with a mixture of TLR ligands. This mixture of TLR ligands was composed of LPS, R848, and poly(I.C) and the cells were stimulated for 2 h. PBS stimulation was used as a control.

We compared the immune responses obtained from each group of individuals by labeling the stimulated leukocytes with the same mass cytometry panel of 33 cell markers. After the acquisition and stimulation of blood samples, a new SPADE analysis was performed using the entire dataset of the cytometry profiles. The SPADE analysis was parameterized to identify 100 cell populations, which was used to perform an automatic gating strategy (Supplementary Figure 2). The SPADE clustering was based on the levels of CD3, CD11a, CD11b, CD11c, CD14, CD16, CD19, CD23, CD28, CD32, CD64, CD66, CD86, CD123, CCR5, CXCR4, Granzyme B, Perforin, TLR2, and HLADR. Cell clusters were annotated according to the expression of CD3, CD11c, CD14, CD16, CD19, CD64, CD66, CD123, and HLADR to identify T-cells, B-cells, neutrophils, NK cells, monocytes, and dendritic cells. These cell populations were isolated for an independent analysis. Then we determined the percentage of cells producing cytokines for each cell type.

**Figure 4:**
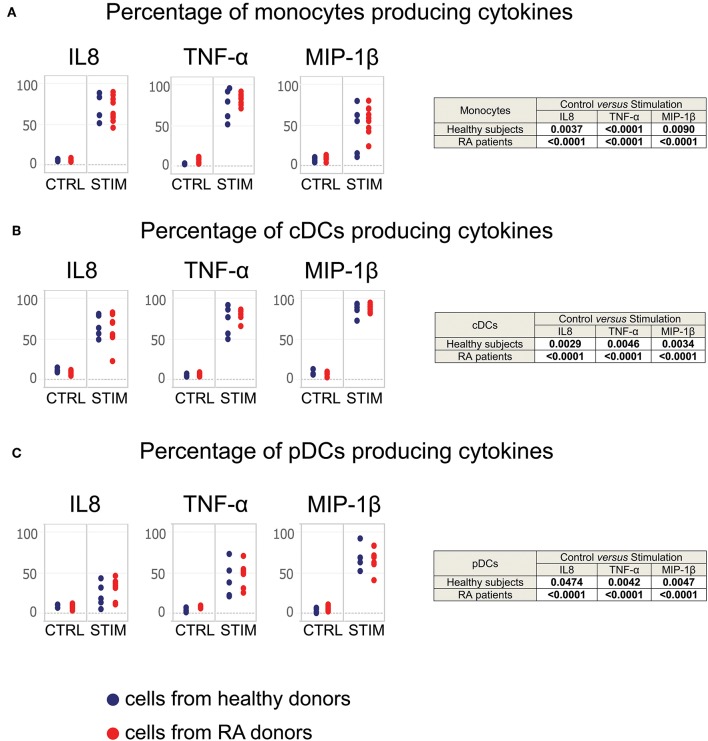
Monocytes, cDCs, and pDCs from RA patients respond normally to TLR stimulation. Whole blood cells from healthy donors and RA patients were stimulated for 2 h with a mixture of TLR ligands. Controls were performed using PBS. The staining of cytokines was performed intracellularly. Monocyte, cDC, and pDC populations were exported from the SPADE analysis represented in Supplementary Figure 1. The percentage of **(A)** monocytes, **(B)** cDCs, and **(C)** pDCs producing MIP-1β, TNF-α, and IL-8 after stimulation are presented. Blue points correspond to the percentages of cells obtained from healthy subjects, red points to those from RA patients.

We did not observe cytokine production by T-cells, B-cells, NK cells, neutrophils, or basophils from either the RA patients or healthy donors. Conversely, we detected the production of cytokines by monocytes, conventional dendritic cells, and plasmacytoid dendritic cells after TLR stimulation (Figure 4). Therefore, we focused our analysis on these three populations.

After 2 h of stimulation, both monocytes from RA patients and healthy donors significantly produced IL-8, MIP-1β, and TNF-α (Figure 4A), as did conventional dendritic cells and plasmacytoid dendritic cells from all subjects (Figures 4B,C). There were no significant differences in the percentages of cells from RA patients and healthy donors.

Thus, leukocytes from RA patients appear to respond normally to TLR stimulation, suggesting that RA does not lead to cell exhaustion in treated patients.

## Discussion

Here, analysis of leukocyte populations by mass cytometry and a set of 33 markers showed that the neutrophil population is much more diverse in RA patients than controls. Among neutrophils, we identified a potential novel RA-specific subpopulation, which was characterized by the CD11b^low^ CD16^high^ phenotype and positively correlates with disease duration. We also identified a potential novel RA-specific T-cell subpopulation, which displayed a CD11a^high^ Granzyme B^high^ phenotype and tended to be negatively correlated with disease activity. Finally, upon TLR stimulation, the cell subsets from both RA patients and healthy donors responded similarly.

Neutrophils are the most abundant cells of the immune-cell population, constituting between 50 and 60% of all circulating leukocytes. In our study, 46.6% of clusters generated by the SPADE analysis corresponded to those of neutrophils. This population displayed a high degree of diversity. Moreover, more than half of neutrophil clusters were associated with RA (but not necessarily significantly), suggesting that RA further increases neutrophil diversity.

The capacity of neutrophils to quickly produce TNF-α, IL-1α, IL-6, and IL-8 after LPS stimulation has already been described in sorted cells (10). However, both R848 and LPS stimulations induced here a production of cytokines only in monocytes and dendritic cells. We hypothesis that red blood cells or other leukocytes populations are interfering here with the ability of neutrophils to respond to LPS stimulation.

The identified potentially new RA-specific subpopulation of neutrophils displays a CD11b^low^ and CD16^high^ phenotype. Importantly, the functions of these markers are key in the pathogenesis of RA. CD11b is known as integrin alpha M and is involved in the adhesion and migration of neutrophils, two features that are required to move from the blood to the affected joint. Steroid treatment has been previously shown to reduce CD11b mean fluorescence intensity in RA-patient neutrophils (11). Although the effect of other treatments on CD11b expression has not been explored, the lower level of CD11b on neutrophils from our patients relative to that of controls could be linked to treatment, since all of our patients were treated at the time of blood sampling. However, such a reduction was not linked to lower disease activity. Moreover, as the decrease of CD11b on neutrophils was associated with an increase of CD16, this reduction could indicate the presence of a potential new subset of neutrophils, possibly involved in the pathogenicity of the disease.

Patients with RA show an increased number of CD14^bright^ CD16^+^ monocytes, called “intermediate monocytes” (4). However, there is no previous description of CD16 upregulation on neutrophils of RA patients. The upregulation of CD16 by neutrophils could have a strong impact, as it could enhance both the degranulation of inflammatory mediators and cytotoxic effects (12).

We identified a potentially new CD11a^high^ Granzyme B^high^ T-cell subpopulation that is specifically enriched in RA patients. Not all important T-cell characterization markers were included (such as CD4 and CD8), as the mass cytometry panel that we used was designed to focus on innate immunity. The absence of the CD4 and CD8 markers in the cytometry panel is a limitation to this study. However, based on the phenotype of this T-cell subpopulation and its abundance among all CD3^+^ T-cells, we suggest that this CD11a^high^ Granzyme B^high^ T-cell subpopulation mainly corresponds to CD8^+^ T-cells. Indeed, few circulating CD4+ T lymphocytes express Granzymes A or B. In addition, granzyme B^+^ CD4^+^ T cells are described as being memory T cells in low abundances (13, 14).

Interestingly, CD11a has been shown to regulate effector CD8 T-cell differentiation and contributes to the primary CD8 T-cell response in mice in response to infection (15). It is not surprising then that this subset expresses high levels of Granzyme B, which is a feature of differentiated CD8 T-cells. The increase of the CD11a^high^ Granzyme B^high^ subset in RA patients could be a marker of low activity, as there was a trend in favor of an inverse correlation between the size of this subpopulation and disease activity assessed by DAS 28. Indeed, CD8-T-cells have been previously shown to negatively correlate with disease activity in RA synovial fluid and these cells expressed high levels of PD-1 (16). However, PD-1 was not part of our panel and thus we cannot confirm that this CD11a^high^ Granzyme B^high^ subset represents exhausted cytotoxic T-cells.

The fixation method that we used here has been previously applied to profile leukocyte populations from humans and non-human primates in different disease and vaccination models (8, 17, 18). However, neither CD11a^high^ Granzyme B^high^ T-cell or CD11b^low^ CD16^high^ neutrophil subpopulations were previously identified in these studies. Therefore, we believe that the presence of these potential new cell subpopulations is not due to the fixation method.

The dataset that we compiled here is composed of 28 mass cytometry profiles consisting each of 33 cell markers. The confirmation of our results by another single-cell technique, such as flow cytometry, would have been a plus. To compensate for this limitation, public cytometric datasets were explored. Unfortunately, we did not find any cytometry dataset with CD11b and CD16 markers characterizing neutrophils or T-cells from patients having RA disease, or in other types of inflammatory or non-inflammatory arthritis. However, in a recent report focusing on synovial T-cells studied by various techniques, including mass cytometry and single-cell transcriptomic analysis, it was shown that CD8 T-cell subsets are characterized by a distinct granzyme expression pattern (19). Among them, subgroup SC-T5 expressed a high level of granzyme B and was present at the same level in patients with RA and osteoarthritis, a non-inflammatory disease, suggesting that they may be associated with a non-inflammatory state. Moreover, exploration of these data also showed that subgroup SC-T5 also expresses CD11a. Thus, these results support that the CD11a^high^ Granzyme B^high^ T-cell subpopulation identified here probably corresponds to CD8^+^ T-cells.

We previously performed the equivalent data acquisition and phenotypic analyses with samples from viremic and non-viremic HIV-infected patients (17), as these two diseases induce chronic inflammation. Both HIV infection and RA induced the generation of a specific neutrophil subpopulation. However, the phenotypes of these two specific neutrophil subpopulations were different. Indeed, unlike RA patients, for whom the specific neutrophil subpopulation was CD11b^low^ CD16^high^, the neutrophils from HIV patients were CD11b^high^ CD16^high^. Thus, we suggest that the upregulation of CD11b is not systematically observed in situations of chronic inflammation, which reinforces the hypothesis of a marker of specific subpopulations.

Persistent inflammation can lead to immune-cell exhaustion, which would make patients more susceptible to infectious disease (17). We studied the effects of RA on the ability of leukocytes to respond to TLR stimulation, as RA induces persistent inflammation, even after treatment. Contrary to what we found in HIV patients (17), we observed no difference between the ability of leukocytes from RA patients and those from healthy donors to produce cytokines after TLR engagement. Thus, it appears that both RA and the various commonly used treatments have only a weak influence on the immune responses triggered following the detection of infectious pathogens in innate cells.

In conclusion, we were able to use mass cytometry to identify a blood subpopulation of RA-specific neutrophils that express high levels of CD16 and low levels of CD11b, possibly playing a role in the pathophysiology of the disease This subpopulation needs to be characterized better at the transcriptomic and functional level. It should be studied in a larger number of patients to better clarify its association with clinical features. Finally, it could be very informative to assess whether this subpopulation could be a biomarker predictive of the response to anti-GM-CSF or whether it may decrease after treatment with GM-CSF. Other studies are also necessary to clarify the exact phenotype of the CD11a^high^ Granzyme B^high^ T-cell subset in terms of exhaustion markers and confirm its possible association with low disease activity.

## Materials and Methods

### Ethics

All patients gave informed consent and the study was approved by our local ethics committee (protocol n°PP 15-004).

### Blood Collection

Whole blood samples from healthy donors and RA patients were collected in lithium heparin tubes by the Etablissement Français du Sang (EFS, Hôpital Saint Louis, Paris, France) and the Hôpital du Kremlin Bicêtre, respectively.

### Stimulation, Fixation, and Storage

Fresh whole blood samples were stimulated for 2 h at 37°C and 5% CO_2_ in 50-mL plastic tubes (BD Biosciences) with a mixture of 1 μg/ml LPS (Invivogen), 3.14 μg/ml R848 (Invivogen), and 100 μg/ml Poly(I:C) (Invivogen). Brefeldin A (Sigma-Aldrich) in dimethyl sulfoxide (Sigma-Aldrich) was added after 1 h of stimulation to a final concentration of 1 μg/ml. Stimulations were stopped by the addition of a fixation mixture (FM). For 1 ml of blood, 10 ml of FM was used. FM was composed of 36% paraformaldehyde (VWR BDH Prolabo) and contained 18.5% glycerol (Sigma-Aldrich) in 1X-Dulbecco's phosphate buffered saline (DPBS), without CaCl_2_ or MgCl_2_, pH 7.4 (Gibco by Life Technologies). After an incubation of 10 min at 4°C, samples were centrifuged at 800 × g for 5 min at room temperature (RT). Red blood cells present in the pellets were lysed by adding 10 ml Milli-Q water (and by pipetting) at RT for 20 min. After two washes with 1X DPBS (centrifugation at 800 × g for 5 min at RT), cells were counted and distributed in 200-μl aliquots containing 3 × 10^6^ cells. Cells were stored at −80°C in FM.

FM used to fix and store the cells was prepared the day before the experiment and conserved at 4°C. This solution allowed freezing and recovery of all blood leukocytes, especially polymorphonuclear cells, which are highly labile and cryopreservation sensitive (8).

### Staining Protocol

For each sample, 3 × 10^6^ cryopreserved fixed cells were washed twice with staining buffer [PBS/0.5% BSA (Sigma-Aldrich)] and labeled with conjugated antibodies according to the following procedures. Cells were incubated at 4°C for 30 min with a mixture of the metal-labeled surface antibodies (Abs) in staining buffer. Markers stained extracellularly are indicated in Supplementary Table 1. After two washes with 1X DPBS, cells were incubated in fixation solution [PBS/0.6% PFA (Electron Microscopy Sciences Hartfield)] at RT for 20 min and permeabilized with 1X Perm/wash buffer (BD Biosciences) at RT for 10 min. Staining with metal-labeled intracellular Abs and an iridium nucleic acid intercalator in 1X Perm/Wash was carried out as for extracellular staining. Markers stained intracellularly are indicated in Supplementary Table 1. Cells were stored overnight with 0.1 μM iridium nucleic acid intercalator in fixation solution. The following day, cells were washed with Milli-Q water, resuspended in 1 ml Milli-Q water and filtered using a 35-μm nylon mesh cell strainer (BD Biosciences), before the addition of EQ Four-Element Calibration Beads (Fluidigm), according to the manufacturer's instructions.

### Sample Acquisition

Acquisition of each sample was manually performed two times in succession on a CyTOF-1 instrument (Fluidigm). Panels, antibody concentrations, clone names, and antibody tags of all antibodies are shown in Supplementary Table 1. The acquisition of patient samples was performed at different days. Each acquisition consisted of leukocytes from both healthy and RA patients. In addition, an independent quality control of the cytometer was performed before each acquisition.

### Cytometry Data Processing

Cytometry data were acquired using EQ Four-Element Calibration Beads, normalized using Rachel Finck's MATLAB normalizer (20) and concatenated using the FCS file concatenation tool (Cytobank). SPADE analyses and viSNE representations were performed on the Cytobank platform (Mountain View, CA). All SPADE analyses were parameterized with a down-sampling of 5%. viSNE representations were generated based on the Barnes-Hut implementation of the t-SNE algorithm using an equal number of cells for each FCS file. Tableau software was used to display viSNE representations. Cytobank was also used to determinate the percentages of cells producing cytokines. Categorical heatmap representations were generated using the SPADEVizR package (21).

### Statistical Analyses

Statistical analyses related to cell population abundances and the percentages of cells producing cytokines were performed using R, based on a non-parametric permutation test (22). Correlations between the results and clinical data were performed using R, based on a Spearman test.

## Data Availability Statement

Cytometric profiles collected in this study are available in the FlowRepository database under the accession number FR-FCM-Z293.

## Ethics Statement

The studies involving human participants were reviewed and approved by Local ethics committee of the Kremlin Bicêtre hospital (protocol n°PP 15-004). The patients/participants provided their written informed consent to participate in this study.

## Author Contributions

AL, AC, RL, XM, and NT: conceptualization and methodology. AL, SB, AP, XM, and NT: formal analysis and investigation. AL, XM, and NT: resources. AL and NT: writing—original draft. AL, SB, AP, GN, EM, AC, RL, XM, and NT: validation and writing—review and editing. AC and RL: funding acquisition. AC, RL, XM, and NT: supervision.

### Conflict of Interest

The authors declare that the research was conducted in the absence of any commercial or financial relationships that could be construed as a potential conflict of interest.
