# Supplementary material for: Characterization of Phenotypes and Functional Activities of Leukocytes From Rheumatoid Arthritis Patients by Mass Cytometry
Source: Front Immunol. 2019 Oct 18;10:2384. doi: 10.3389/fimmu.2019.02384 (PMC6813461; doi:10.3389/fimmu.2019.02384)
Supplement: Supplementary file 1 [file Data_Sheet_1.PDF]

Characterization of Phenotypes and Functional Activities of Leukocytes from Rheumatoid Arthritis Patients by Mass Cytometry.

Supplementary Materials

Adrien Leite Pereira[1], Samuel Bitoun[1,2,3], Audrey Paoletti[2,3], Gaetane Nocturne[2,3], Ernesto Marcos Lopez[1], Antonio Cosma[1], Roger Le Grand[1], Xavier Mariette[1,2,3,‡], and Nicolas Tchitchek[1,‡,*]

[1]CEA – Université Paris Sud 11 – INSERM U1184, Immunology of viral infections and autoimmune diseases, IDMIT Infrastructure, 92265 Fontenay-aux-Roses, France

[2]Department of Rheumatology, Hôpital Bicetre, Le Kremlin-Bicetre, France.

[3]Center for Immunology of Viral Infections and Autoimmune Diseases, INSERM U1184, Paris-Sud University, Le Kremlin-Bicêtre, France.

[*]Corresponding author: CEA – Université Paris Sud 11 – INSERM U1184, Immunology of viral infections and autoimmune diseases, IDMIT Infrastructure, 92265 Fontenay-aux-Roses, France. Phone: +33146548284. E-mail: nicolas.tchitchek@cea.fr

[‡]These authors contributed equally to the work

Label	Antibody	Clone	Staining	Functions
141Pr	CD66	TET2	Extracellular	Phenotype
142Nd	HLADR	L243 (G46-6)	Extracellular	Activation<br>Costimulation<br>Phenotype
143Nd	CD3	UCHT1	Extracellular	Phenotype
144Nd	CD64	10.1.1	Extracellular	Activation<br>Costimulation<br>Fc receptor
146Nd	IL-6	MQ2-13A5	Intracellular	Mediators
147Sm	CD123	7G3	Extracellular	Phenotype
148Nd	IL4	7A3-3	Intracellular	Mediators
149Sm	CD11a	HI111	Extracellular	Activation<br>Costimulation<br>Adhesion
150Nd	CD11b	ICRF44	Extracellular	Activation<br>Costimulation<br>Adhesion
151Eu	IL-8	NAPII	Intracellular	Mediators
152Sm	CD16	B73.1	Extracellular	Activation<br>Costimulation<br>Fc receptor<br>Phenotype
153Eu	CD23	M-L233	Extracellular	Fc receptor
154Sm	CD86	2331 (FUN-1)	Extracellular	Activation<br>Costimulation
155Gd	CD32	2E1	Extracellular	Fc receptor
156Gd	MIP-1β (CCL4)	D21-1351	Intracellular	Mediators
158Gd	IP10	6D4	Intracellular	Mediators
159Tb	TNF-α	MAb11	Intracellular	Mediators
160Gd	IL-1α	364/3B3-14	Intracellular	Mediators
161Dy	Perforine	DTA G9	Intracellular	Mediators
162Dy	IL-12	C8.6	Intracellular	Mediators
164Dy	CD184 (CXCR4)	12G5	Extracellular	Homing
165Ho	TLR2	REA109	Extracellular	Sensing
166Er	CD195 (CCR5)	3A9	Extracellular	Homing
167Er	CD28	CD28.2	Extracellular	Activation<br>Costimulation
168Er	CD11c	B-ly6	Extracellular	Activation<br>Costimulation<br>Adhesion<br>Phenotype
169Tm	IFN-α	LT27:295	Intracellular	Mediators
170Er	CD14	M5E2	Extracellular	Sensing<br>Phenotype
171Yb	IL10	JES3-9D7	Intracellular	Mediators
172Yb	TLR7	IMG4G6	Intracellular	Sensing
173Yb	Granzyme B	GB11	Intracellular	Mediators
174Yb	CD19	HIB19	Extracellular	Phenotype
175Lu	IL1-RA	1H5	Intracellular	Mediators
176Yb	NFkB-pS529	K10-892.12.50	Intracellular	Activation<br>Costimulation
191/193Ir	DNA Intercalator	-	Intracellular	-

Supplementary Table 1. Antibodies and cell markers used to stain cells from healthy donors and treated RA patients. The metal isotopes, antibody clones, stainings, and the functions of the antibodies used are indicated for each marker.

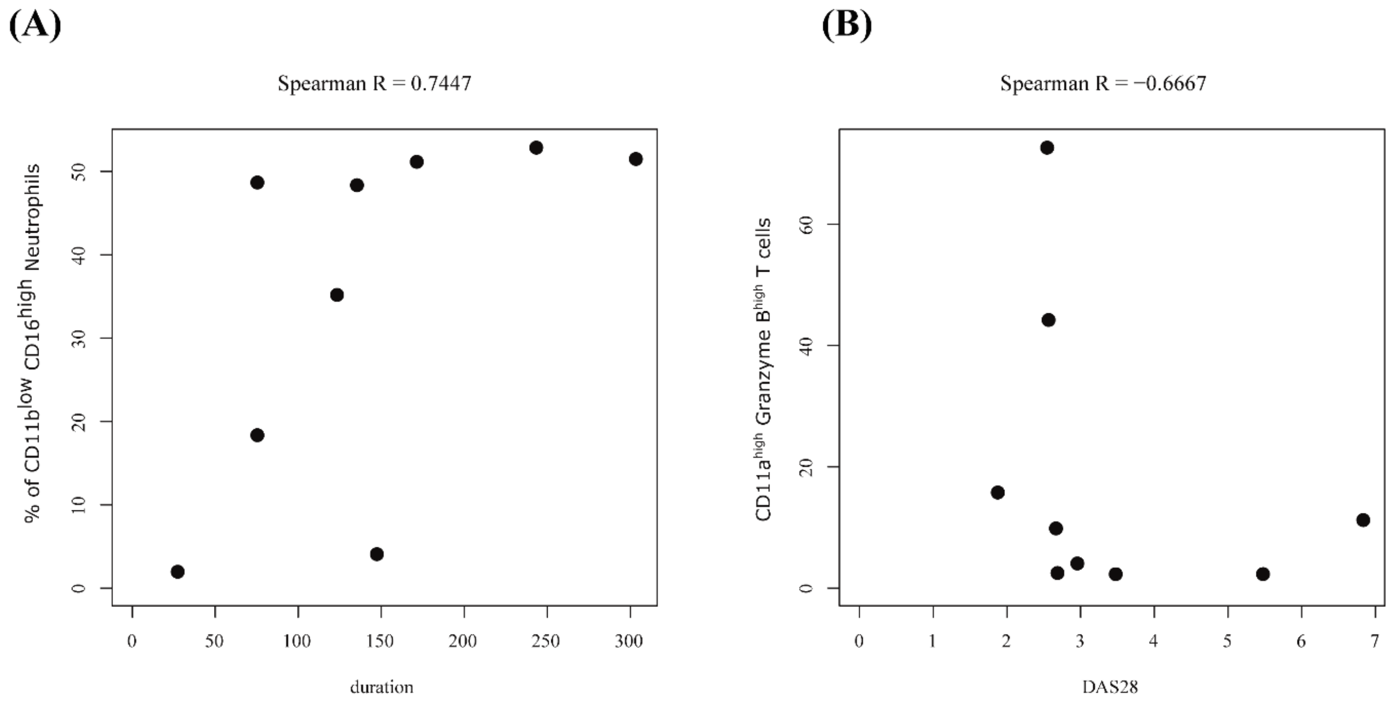

Supplementary Figure 1. Associations between cell population abundances and clinical factors. (A) Dot plot representation showing the correlation between the disease duration and percentage of CD11b^{low} CD16^{high} neutrophils among the entire neutrophil population. **(B)** Dot plot representation showing the correlation between the DAS28 and the percentage of CD11a^{high} Granzyme B^{high} T-cells among the entire T-cell population. Correlations were determined based on the Spearman coefficient of correlation.

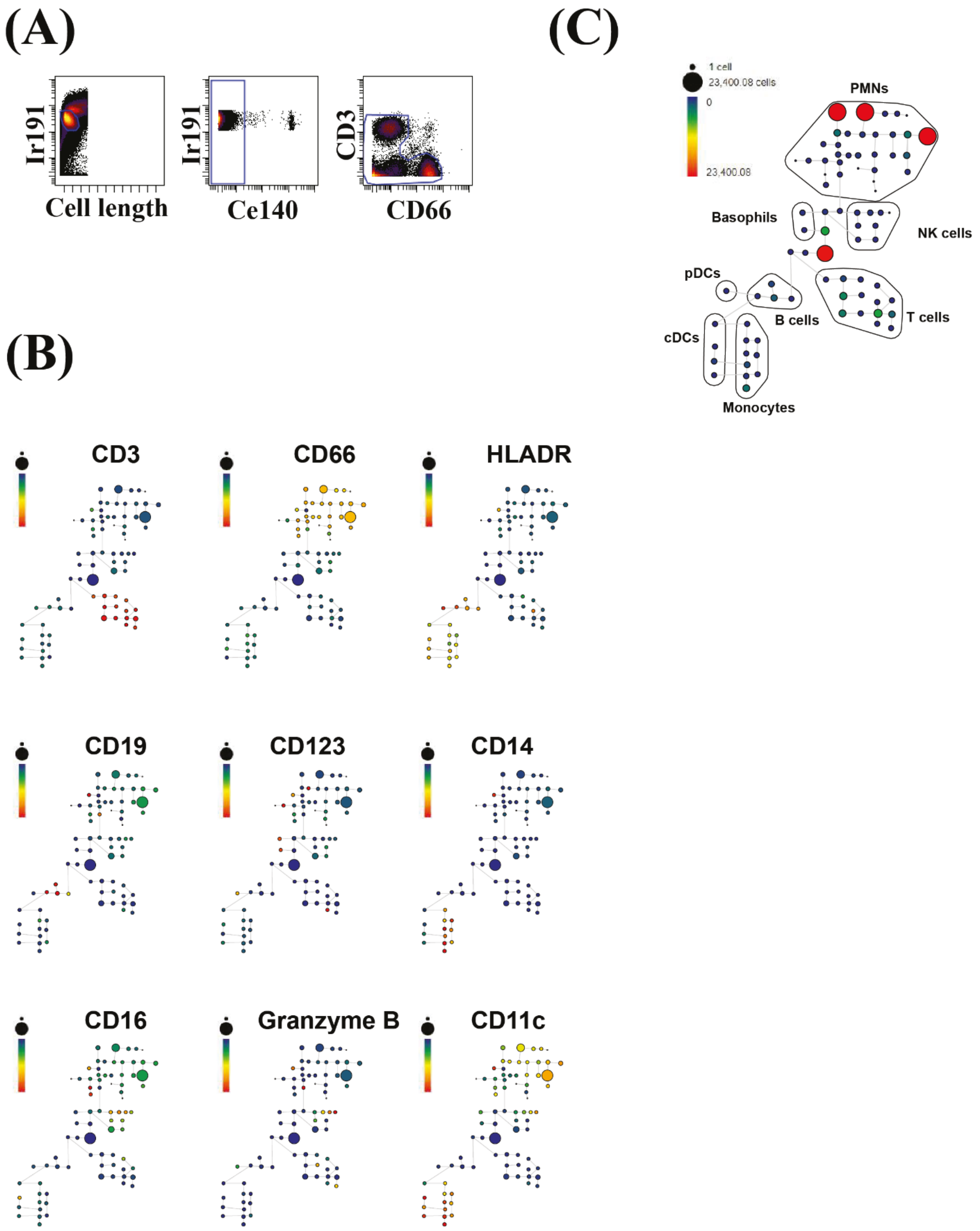

Supplementary Figure 2. Annotation of cell clusters identified by SPADE. (A) Samples were manually gated to exclude the EQTM Four-Element Calibration Beads, select singlets, and gate out nonspecific background generated by metal conjugated Ab-binding eosinophils. **(B)** SPADE analysis was performed to identify 100 cell clusters using a 5% down-sampling parameter. The clustering was based on the levels of CD3, CD11a, CD11b, CD11c, CD14, CD16, CD19, CD23, CD28, CD32, CD64, CD66, CD86, CD123, CCR5, CXCR4, Granzyme B, Perforin, TLR2, and HLADR. The median expression of CD3, CD11c, CD14, CD16, CD19, CD64, CD66, CD123, and HLADR were overlaid on the generated SPADE tree to annotate each cluster. **(C)** Eight leukocyte populations have been identified based on the median expression of these specific markers. Neutrophil clusters were designated as CD66^{+}, T-Cells clusters as CD3^{+}, NK cells clusters as HLADR^{-} CD16^{+}, basophil clusters as HLADR^{-} CD123^{+}, B-cell clusters as HLADR^{+} CD19^{+}, monocyte clusters as HLADR^{+} CD14$^{+/-}$ CD64^{+}, conventional dendritic cell (cDCs) clusters as HLADR^{+} CD11c^{+} CD14^{-} CD64^{-}, and plasmacytoid dendritic cell (pDCs) clusters as HLADR^{+} CD123^{+}.
